# Accelerated TMS - moving quickly into the future of depression treatment

**DOI:** 10.1038/s41386-023-01599-z

**Published:** 2023-05-22

**Authors:** Sanne J. H. van Rooij, Amanda R. Arulpragasam, William M. McDonald, Noah S. Philip

**Affiliations:** 1 Emory University School of Medicine, Department of Psychiatry and Behavioral Sciences, Atlanta, GA USA; 2 Alpert Medical School of Brown University, Department of Psychiatry and Human Behavior, Providence, RI USA; 3 VA RR&D Center for Neurorestoration and Neurotechnology, VA Providence Healthcare System, Providence, RI USA

**Keywords:** Outcomes research, Depression, Depression

## Abstract

Accelerated TMS is an emerging application of Transcranial Magnetic Stimulation (TMS) aimed to reduce treatment length and improve response time. Extant literature generally shows similar efficacy and safety profiles compared to the FDA-cleared protocols for TMS to treat major depressive disorder (MDD), yet accelerated TMS research remains at a very early stage in development. The few applied protocols have not been standardized and vary significantly across a set of core elements. In this review, we consider nine elements that include treatment parameters (i.e., frequency and inter-stimulation interval), cumulative exposure (i.e., number of treatment days, sessions per day, and pulses per session), individualized parameters (i.e., treatment target and dose), and brain state (i.e., context and concurrent treatments). Precisely which of these elements is critical and what parameters are most optimal for the treatment of MDD remains unclear. Other important considerations for accelerated TMS include durability of effect, safety profiles as doses increase over time, the possibility and advantage of individualized functional neuronavigation, use of biological readouts, and accessibility for patients most in need of the treatment. Overall, accelerated TMS appears to hold promise to reduce treatment time and achieve rapid reduction in depressive symptoms, but at this time significant work remains to be done. Rigorous clinical trials combining clinical outcomes and neuroscientific measures such as electroencephalogram, magnetic resonance imaging and e-field modeling are needed to define the future of accelerated TMS for MDD.

## Introduction

One of the most challenging clinical problems in the acute management of mood disorders has been the delay in treatment response. While much of the focus has been on increasing the efficacy of antidepressant treatments, another critical problem is that first-line treatments such as pharmacotherapy and psychotherapy typically do not produce significant symptom improvements for weeks at a time. Neuromodulation strategies which target neural circuits directly may provide a more direct and faster effect on depressive symptoms. Therapeutic Transcranial Magnetic Stimulation (rTMS, hereafter referred to as TMS for simplicity across acronyms) has been FDA-cleared for pharmacoresistant major depressive disorder (MDD) for over a decade and is routinely used in clinical practice [1, 2]. TMS is typically delivered using 10 Hz over the left dorsolateral prefrontal cortex (l-DLPFC) once a day initially for 37.5 min over a 6-week period, although recent adjustment to the interstimulus interval can reduce a single session to ~20 min [3]. In its current iteration, TMS is limited by a slow response time and may pose additional challenges for patients working full time or with transportation or childcare concerns, given the need for daily administrations. Slow treatment response time is especially concerning in patients with acute, debilitating symptoms including suicidal thoughts and can decrease treatment compliance and increase morbidity. Thus, the field has identified a clear need to accelerate treatment response.

Accelerated TMS, defined as a protocol delivering more than one daily TMS session, is one emerging delivery schedule of TMS aimed to reduce treatment duration and improve response time, with the goal of achieving similar (or superior) levels of efficacy. Recently, the FDA cleared an accelerated TMS protocol for depression, i.e., the Stanford Neuromodulation Therapy (SNT; formerly Stanford Accelerated Intelligent Neuromodulation Therapy, SAINT) protocol which consists of five days of 10 sessions of intermittent theta burst stimulation (iTBS) per day. The focus of this review will be to examine the evidence of accelerated TMS, including the SNT protocol, in the treatment of MDD. MDD was selected because it has the most extant data, and the insights gained from this research may inform research related to other neuropsychiatric disorders.

In this piece, we first review lessons learned from attempts to accelerate other treatments for depression and make the case for accelerated TMS. Then we discuss the evidence for accelerated TMS as well as critical components to consider for accelerating the treatment response: treatment parameters, i.e., stimulation frequency and inter-stimulation interval, cumulative exposure, i.e., number of treatment days, sessions per day and pulses per session, individualized parameters, i.e., treatment dose and target, and brain state, i.e., context and concurrent treatments. The promise and pitfalls of accelerated TMS is reviewed, and the review ends with synthesis and highlights several key areas needed to move the field forward in this area.

## Lessons learned from accelerating other treatments

The concept of accelerating antidepressant treatments long preceded the discussion of accelerating TMS. As early as 1969, Arthur Prange recognized the importance of “enhancing” the efficacy of imipramine by adding thyroid hormone (L-triiodothyronine or T_3_) which was hypothesized to increase receptor sensitivity due to the interaction of the neuroendocrine system with depression [4]. In a pivotal study, Prange’s group demonstrated both a decrease in morbidity and duration of hospitalization in patients on imipramine augmented with T_3_. Subsequent studies have supported strategies to improve the antidepressant response using sleep deprivation or the addition of L-triiodothyronine, lithium, atypical antipsychotics, or pindolol to antidepressant monotherapy [5]. However, Altschuler et al. [5] pointed out that these studies were limited by small sample sizes as well as failures to provide an a priori definition of a shorter or *accelerated* response time. In fact, most of these early studies focused on an improved antidepressant response, often in patients who had failed monotherapy, rather than a shortened time to response.

For the most commonly used antidepressant medications (e.g., tricyclics (TCA’s), serotonin selective reuptake inhibitors (SSRI’s)), patients typically do not show a significant decrease in depressive symptoms for 3–6 weeks. This delay in the antidepressant response occurs in spite of the fact that the neurophysiological effect associated with antidepressant potency, the blockade of the membrane neurotransmitters transporters, happens almost immediately after starting the medication [6]. The delay is hypothesized to be related to the time it takes for the neuroplastic downregulation of post-synaptic serotonin (5-HT) and noradrenergic receptors and desensitization of autoreceptors located on 5-HT and noradrenergic cell bodies [6]. Other theories have cited evidence that the neurochemicals in the neurotropic signaling cascade (e.g., cyclic adenosine monophosphate, brain-derived neurotrophic factor, bcl-2, and mitogen activated protein kinases) are critical to the antidepressant response and again there is a delay in neurophysiologic changes in synaptic connections that restore critical brain circuits [7]. The addition of psychotherapy to antidepressant medication has been shown to improve remission rates in treatment-resistant depression (TRD) and decrease relapse and recurrence [8–10]. However, psychotherapy has not been shown to accelerate the time to response in MDD either as a monotherapy or in combination with somatic treatment.

Intravenous ketamine has a very different mechanism of action from the traditional antidepressants which exert their antidepressant effect through the monoamine system. Ketamine, an N-methyl-D-aspartate (NMDA) receptor antagonist, acts through amino acid neurotransmitters (i.e., ƴ-aminobutyric acid (GABA) and glutamate) [11]. As a glutamate receptor modulator, ketamine acts as a non-competitive channel blocker of the NMDA receptors on inhibitory GABA neurons. This blockade results in a glutamate surge which activates 2-amino-3- (5-methyl-3-oxo-1,2-oxazol-4yl) propanoic acid (AMPA) receptors leading to elevated levels of brain-derived neurotrophic factor (BDNF) and phosphorylation of tropomyosin receptor kinase B (TrkB) as well as potential downstream effects on the mammalian target of rapamycin (mTOR) pathway [12]. The mechanism of action of ketamine likely involves the opioid system, as demonstrated by a study where an opiate receptor antagonist was able to block antidepressant effects of ketamine [13]. This increase in neuroplasticity may have a more immediate effect than the action of SSRIs and TCAs on depressive symptoms. The evidence from a recent Cochrane analysis is that both ketamine and its s-enantiomer, esketamine, demonstrate efficacy in treating depressive symptoms over placebo within 24 h [12]. However, there are significant concerns about the durability of the ketamine response with patients often requiring extended maintenance sessions to sustain the antidepressant effect. There are also the potential long term side effects of maintenance treatment with ketamine including tolerance, dependence, and cognitive side effects [9, 14].

Psychedelics (e.g., psilocybin assisted psychotherapy), have evidence for efficacy in depression after only one or two sessions and this antidepressant efficacy is durable in many patients [15]. Putative mechanisms involving activation of 5-HT2A receptors are thought to underlie this antidepressant effect [16, 17]. The initial evidence for efficacy led to the FDA designating psilocybin as a “breakthrough therapy” for TRD. While psilocybin assisted psychotherapy holds promise for continued investigation, the use of psilocybin in depression more broadly has only begun to be examined [18].

The gold-standard non-pharmacological treatment for depression is electroconvulsive therapy (ECT), which has been shown to have a significant and relatively immediate effect on depressive symptoms and suicidal ideation via a generalized seizure, albeit in a minority of TRD patients [19, 20]. However, the majority of patients treated with ECT do not respond for 6–8 treatments given over 3-4 weeks. Attempts to accelerate the ECT response using multiple treatments in one day have resulted in unacceptable side effects primarily to detrimental effects on cognition [21, 22].

With the advent of advanced neuromodulation techniques, neural circuits may potentially be more precisely targeted to effect change earlier in the treatment course. Unfortunately, more precise targeting has not always been associated with an accelerated treatment response. The most precise neuromodulation technique, deep brain stimulation (DBS) [23], which targets a few millimeters of subcortical tissue, is associated with a response time of months which is similar to less precise neuromodulation methods such as vagal nerve stimulation (VNS) [24]. Notably, ventral tegmental area (VTA) stimulation has been associated with rapid effects in MDD [25], though it should be noted that ECT, DBS and VNS typically are only used in TRD patients and symptom severity, and treatment resistance status may also affect the time to response in this patient population.

Overall, none of the treatments discussed above have achieved an accelerated treatment response in MDD. While some treatments are still under investigation, challenges of accelerating treatment response using pharmacological and invasive brain stimulation techniques include a heightened risk of more or more severe side effects, reduced tolerability, or neurophysiological characteristics of the treatment that prevent successful acceleration.

## The case for accelerated TMS

Two noninvasive neuromodulation techniques with relatively benign side effect profiles are prime candidates to investigate the impact of direct and more precise neural stimulation in accelerating the antidepressant response: transcranial direct current stimulation (tDCS) and TMS. tDCS uses a weak electrical current to provide stimulation via an anode and cathode placed on the scalp to inhibit or facilitate neural circuits to affect cognition, motor skills, psychotic and mood disorders [26]. Functional neuroimaging (e.g., functional Magnetic Resonance Imaging; fMRI) and electroencephalograms (EEGs) have been used to examine the network changes before, after, and during tDCS so perturbations in the networks can be analyzed and potentially enhanced to accelerate the response. tDCS is also relatively inexpensive to administer and could potentially be self-administered by a patient with minimal training, and therefore allow for maximizing the number of stimulations to accelerate treatment. Despite these advantages, the evidence for an antidepressant effect for tDCS is mixed and the optimal parameter settings for treating depression are continuing to develop [27–29].

In contrast, TMS has a number of advantages when reviewing methods to accelerate the antidepressant response. TMS is FDA cleared to treat MDD and has been widely adopted in clinical settings which may facilitate the eventual use of an accelerated protocol in clinical practice. Furthermore, it has a benign side effect profile, is amenable to multiple daily administration, and has neuroimaging data supporting the relationship of the antidepressant effect with neurophysiologic changes (reviewed further below). Accelerating the response time to TMS would also address the burden of time required for daily administrations over weeks (and associated staffing requirements). Given these factors, there has been increasing interest in exploring various forms of accelerated TMS in which multiple TMS sessions are delivered per day.

## Core elements of accelerated TMS

**Fig. 1:**
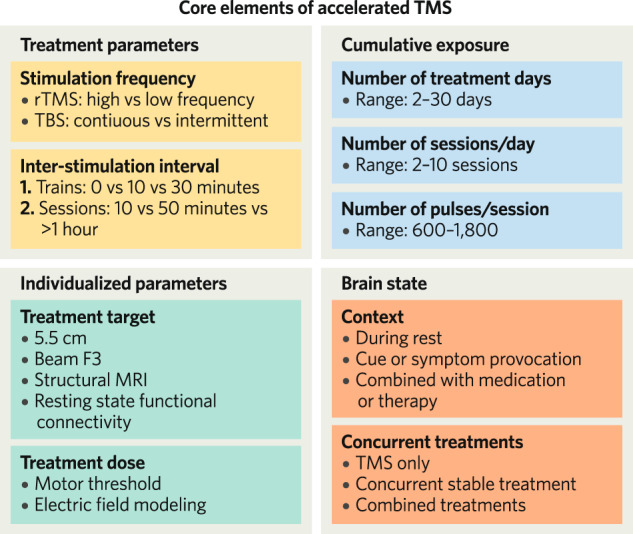
Core elements of accelerated TMS. The nine core elements considered in this review for accelerated TMS. rTMS repetitive transcranial magnetic stimulation, TBS theta-burst stimulation, MRI magnetic resonance imaging.

Preliminary evidence suggests that a more rapid improvement in depressive symptoms may be achieved with accelerated TMS protocols. However, the accelerated TMS protocols in clinical trials have not been standardized and vary considerably across a variety of elements. In this section we discuss available data on the core elements to be considered in an accelerated TMS protocol (Fig. 1).

### Treatment parameters

TMS treatment parameters vary between protocols and stimulation frequency and inter-stimulation interval are essential elements to consider for accelerating TMS.

#### Stimulation frequency

TMS stimulation protocols have utilized a variety of frequencies. TMS is typically defined as either high frequency (at least 5 Hz) or low frequency (most often 1 Hz), and are typically designated to be excitatory or inhibitory [30–33], respectively, to cortical neural transmission. The relationship of TMS frequency to neuronal firing arose from studies of corticospinal excitability applying TMS to the motor cortex (reviewed in ref. [34]). This observed directional relationship was later corroborated by positron emission tomography or single photon computed emission tomography studies [35–37]. Some comparable results were observed in early studies of TMS applied over the DLPFC [38]. That stated, this simple binary heuristic likely represents a dramatic oversimplification; neuroimaging studies of TMS, particularly using functional connectivity measures, have not consistently replicated this directionality [34–36].

One practical impediment to increasing the number of sessions per day is the length of the TMS sessions (20–40 min) needed to deliver high frequency trains of pulses with the necessary intertrain pause. A key development was a technological advancement that significantly decreased the time needed to administer TMS treatments while maintaining the number of pulses per session. In 2005 investigators began to evaluate the safety and efficacy of theta burst TMS stimulation [37]. This stimulation approach, intermittent theta burst TMS or iTBS, was found to mimic endogenous hippocampal theta neural activity and provide short bursts of intermittent stimulation at high frequencies which allowed for the delivery of multiple TMS pulses in a matter of a few minutes.

In 2018, Blumberger et al. [38] provided a key step towards the development of accelerated TMS, by conducting a non-inferiority randomized clinical trial that compared 3000 pulses of TMS with 600 pulses of iTBS per day, each delivered five days per week for 4–6 weeks. This study clearly demonstrated that once-daily iTBS was non-inferior to standard TMS, yet with the critical advantage of shortening the session time from 37.5 to 3 min per treatment session. Thus, the development of iTBS protocols has allowed investigators to administer multiple iTBS sessions a day by increasing the number of sessions a day and is a major contributor to the rapid expansion of accelerated TMS protocols for research and clinical practice.

A different form of TBS, continuous TBS (cTBS), can also be applied in an accelerated form as 600 pulses are delivered in 40 s. The traditional assumption, based on studies of corticospinal excitability is that iTBS may yield LTP-like effects, whereas cTBS may provide LTD-like effects. However, more recent work has clearly demonstrated this is an oversimplification and is likely incorrect (e.g., refs. [39, 40]). One recently published accelerated cTBS pilot study [41] for patients with Obsessive Compulsive Disorder delivered 1800 pulses (600 bursts 3 pulses each) per session, 10 sessions per day for 5 days to the right frontal pole. The study showed preliminary data on safety, feasibility, and efficacy of accelerated cTBS with 71% efficacy and minimal side effects. Of note there is far less data on accelerated cTBS and therefore the remainder of the review predominantly focuses on iTBS.

#### Inter-stimulation interval

As described above, standard TMS protocols typically involve a single session per day with ≥24 h spacing of sessions. Reduction of the inter-stimulation interval would support an accelerated protocol and may enhance the efficacy of certain types of TMS stimulation, such as TBS. There are two types of inter-stimulation intervals to be considered: the time between stimulation trains and the time between sessions. Cole and colleagues [42, 43] have utilized a reduced intertrain interval compared to standard TMS by delivering three consecutive iTBS sessions of 600 pulses per session. Additionally, in this protocol, there is a 50-min interval between treatment sessions which was guided by animal work that has demonstrated that hour-long intervals between sessions may be optimal for producing long term potentiation via TBS [44, 45]. Other studies, however, have used as little as 10-min breaks or as much as 10 h between sessions (reviewed in refs. [46, 47]), and clear data on best parameters is unavailable. A study examining the optimal interval between TMS trains (600 iTBS pulses/session) showed that 1800 pulses with no interval resulted in reduced cortical excitability, whereas 10- and 30-min breaks enhanced cortical excitability, with maximum excitability observed in the 30-min interval group [48].

#### Summary – Protocols and parameters

iTBS allows for more treatment sessions per day, and therefore more flexibility in accelerating TMS treatments. While studies showing efficacy for accelerated iTBS have been published, there are many unknowns and a better understanding of the interplay between stimulation frequency and inter-stimulation parameters is critical. Both the stimulation frequency and inter-stimulation interval may determine whether the TMS stimulus is either excitatory or inhibitory. Investigators are still working to determine the optimal interval. It is likely that a number of individual factors will also influence cortical excitability, including a person’s neuroanatomy, the disease state, complicated by likely non-linear effects with cumulative exposure on MEPs [40]. Individualizing treatments such as real time electroencephalogram (EEG) feedback loops (e.g., ref. [49]) could also inform us about preferred intervals instead of using average data.

### Cumulative exposure

Cumulative TMS exposure or TMS pulses delivered during a treatment course is driven by three main factors: the number of treatment days, the number of treatment sessions per day, and the number of pulses per session.

#### Number of treatment days and the number of sessions per day

The earliest attempts to accelerate the TMS response increased the number of sessions from one to two a day for the treatment of depression [50, 51] and schizophrenia [52] with positive clinical outcomes and tolerability. In an unblinded trial, Holtzheimer et al. [53] delivered 15 high frequency TMS sessions over a two-day period. These sessions were administered (five consecutive hourly sessions on Day 1, and ten consecutive hourly sessions on Day 2) using 10 Hz TMS in 5 s trains with a 25 s intertrain interval at 100% of motor threshold. A significant treatment effect was achieved by day three and maintained six weeks later. Positive clinical and tolerability outcomes have since been observed in protocols delivering two TMS sessions per day [54] as well as five TMS sessions per day [55]. However, in a large recent RCT no significant differences in clinical improvements were observed when comparing twice daily (two times 600 pulses) versus once daily (1200 pulses) iTBS, and response and remission rates were similar in both groups [56], indicating the number of sessions per day (alone) is unlikely to improve treatment outcomes.

A recent review by Caulfield et al summarizes the extant data on studies using accelerated TMS protocols [46]. There was notable variability across the majority of studies; the authors reviewed 63 accelerated TMS studies that administered 2–10 sessions per day for 2–30 treatment days, and the total number of TMS sessions ranged from 9 to 104. This review reported initial evidence that increasing the number of sessions per day, the total number of sessions, and the total number of pulses each appear to have a positive relationship with response rate, which was postulated to be linear. It is important to consider, however, that these parameters are fundamentally interrelated as more sessions per day typically equates to more sessions in total as well as more pulses. It is therefore key to understand the interaction between the treatment parameters and cumulative exposure elements.

The largest number of sessions per day to date has been implemented by Cole and colleagues [42, 43]. The effects of increasing the number of treatment sessions per week was studied by delivering ten sessions of theta burst stimulation per day for five days. The FDA recently cleared the SNT protocol following high remission rates in a small number of severely depressed patients (*n* = 29), where *n* = 11 (78.6%) met MDD remission criterion at some point in their participation, and no serious adverse events [42, 43]. One other study employed a similar protocol and showed significant improvements in post-partum depression [57].

#### Number of pulses per session

Current TMS protocols typically deliver 3000 pulses/day (or 1800 pulses for a 1 Hz TMS) but published accelerated TMS studies have applied a considerably higher number of pulses per day or session. The initial accelerated TMS study led by Holtzheimer and colleagues delivered 7500 pulses per day [53] while additional studies employing 10 Hz TMS delivered up to 6000 pulses/day [54]. Similarly, in another unblinded trial, Hadley et al. [58] provided a 2-week accelerated 10 Hz TMS protocol involving a higher number of pulses (6800 pulses/day) delivered in once daily sessions to patients with TRD. In this study, 33% of patients met criteria for clinical remission at the conclusion of the study and importantly, the higher TMS doses were well tolerated without significant adverse outcomes. The largest reported number of pulses per session is the protocol described by Cole et al. [42, 43]. In this protocol, 1800 pulses are delivered per session for ten sessions per day, totaling 18,000 pulses per day, providing evidence that this high number of pulses with 50-min delays between sessions can be delivered safely.

A notable difference between rTMS and iTBS is the pulse pattern. Repetitive TMS includes delivery of stimulation at a particular frequency (e.g., 10 Hz), whereas during TBS, triplets of high-frequency (50 Hz) stimulation are repeated at 5 Hz (200 ms interval), designed to resemble hippocampal theta oscillations [37]. While a comprehensive comparison of different patterns is outside the scope of this review (these are reviewed in more detail in ref. [46]), future studies are needed to compare whether the pattern itself or the ability of TBS to deliver large numbers of pulses in a short period of time is driving clinical changes.

Sequential bilateral TMS is another novel approach to accelerate or enhance clinical outcomes. While initial studies of bilateral TMS did separate from sham [59], later examinations have not found superiority of bilateral TMS to unilateral TMS [60]. Bilateral theta burst TMS and bilateral repetitive TMS were both shown to be superior to sham TMS in a network meta-analysis of randomized clinical trials [61]. Recent data has indicated that sequential bilateral TMS appears noninferior to bilateral theta burst TMS [62], although whether this yields an accelerated response compared to standard unilateral TMS remains unknown.

#### Summary – Cumulative exposure

Extant studies have shown that delivering as much as ten sessions per day with a total of 18,000 pulses for five consecutive days (90,000 pulses in total) appears safe from early pilot randomized controlled trials. What is not known, however, is if there is value in less than this number of treatments per day. From a practical point of view (clinical hours, staff availability) ten sessions per day seems to be the maximum number of treatments per day given that with treatment time and delay between treatments this would take ten hours to administer. It is unclear if, for example, five sessions per day spread out over two weeks has similar treatment benefits or whether the succinct delivery has biological advantages.

Of note, none of the available data include biological readouts of brain-based response, leaving the best estimates of efficacy to rely upon clinical rating scales. While these are the standard of care, EEG or fMRI obtained during the treatment course is likely to be required to better characterize (and utilize) individual differences in future protocols.

### Individualized parameters

Treatment response can possibly be accelerated by using a more personalized treatment approach to improve efficacy and thereby reduce the number of treatments needed for optimal response. Specifically, treatment dose and treatment target can be individualized using different neuroimaging techniques. In current standard treatment protocols, these parameters are adjusted to the individual using motor threshold testing to define the treatment dose (using 80–120% of the output over the motor strip to typically move the contralateral abductor pollicis), and to locate the TMS target (applying the 5.5 cm rule) or use the Beam F3 method [63] which accounts for individualized head size and shape. In line with ongoing research, we discuss the use of neuroimaging methods in further personalizing and optimizing these parameters.

#### Treatment target

The definition of the TMS treatment target could be individualized using structural and functional MRI. Structural MRI is used to provide details of the neuroanatomy and can be used to locate the structurally defined individualized TMS target. Functional connectivity (FC) networks link neuronal areas to projections on the cortex and TMS is applied to an area on the skull corresponding to the underlying cortex to “target” stimulation of the neuronal structure. Resting state (RS) fMRI scans are typically utilized for FC analyses to define the individual’s TMS treatment targets. FC has been used in accelerated TMS protocols [64–66] to demonstrate that baseline anti-correlation patterns to the subgenual anterior cingulate cortex (sgACC) were reversed in responders after accelerated TMS treatment but not in non-responders. Following the rationale that cortical areas are connected with deeper brain regions through functional networks, MRI connectivity measures may help define the best cortical target that engages the network and the subcortical region of interest.

Cole et al. [42, 43] used RS fMRI scans to define the area within the l-DLPFC (defined as Brodmann Area 46) that has the strongest anticorrelation with the sgACC, following earlier research showing the greatest clinical effect for the TMS target site with the greatest negative correlation [67]. Assessment of personalized DLPFC targeting using RSFC with the sgACC showed substantial individual variability but across time stability in 1000 healthy volunteers with RS scans [68]. These studies suggest precision of this individualized targeting method, however, no direct comparisons between individualized TMS treatment targets and other protocols have been published to date and the added benefit of this method has yet to be confirmed. In fact, some of the few prospective studies comparing neuronavigated TMS versus scalp-based targeting have failed to clearly demonstrate clinical superiority of basic neuronavigation [65]. Moreover, a recent fMRI study [69] estimated sgACC functional connectivity with the stimulation site and related to TMS treatment outcomes in a large number of patients with MDD. Notably, while sgACC functional connectivity with the stimulated area predicted a small degree of the treatment response (2.56% of the variance), this effect was mainly explained by respiratory patterns of a subgroup of patients. Furthermore, predictive models incorporated electrical field modeling, which reduced the spatial precision of the engaged cortex. This study raised serious questions about whether sgACC-dlPFC functional connectivity has sufficient reliability to define personalized TMS treatments; furthermore, how this connectivity intersects with the extensive cumulative exposure of accelerated TMS needs to be carefully examined and considered.

#### Treatment dose

Individualized targeting approaches using FC does not generally account for TMS focality. As proposed by Balderston et al. [70, 71], combining targeting using RSFC with electrical (E) field modeling is another approach with potential to optimize TMS target location and coil orientation. E-field modeling uses an individual’s structural scan to approximate the directionality and amplitude of the induced electrical current in the brain based on the location and orientation of the coil. Balderston’s proposed method reduces interindividual variability in stimulation site and ideal coil orientation and decreases the distance between the scalp and the cortical target. Findings from their proof-of-concept study [70] suggest individualized targeting may maximize clinical efficacy and contribute to predicting treatment response. Other studies are ongoing that aim to optimize the stimulation parameters based on each individual’s cortical electrical field (e.g., ref. [72]). That stated, it should be expected that some individuals may require above the standard 120% of motor threshold, and the safety profile of therapeutic TMS (including iTBS) at suprathreshold intensities remains an important consideration.

#### Summary – Individualization

While the application of individualized TMS treatments is still in its early days, the available data incorporating neuroimaging and targeting appears promising. That stated, whether and how to utilize these approaches, and whether they are clinically superior to standard methods, requires further definitive evidence. As described above, the general paucity of biological readouts or measures to assess the impact of individualization complicates interpretation of the existing data. Yet, these precision approaches are likely to be able to provide the evidence to demonstrate their clinical utility. As addressed below, an important factor to consider for these individualized approaches is access to the technologies needed, especially in clinical settings, as well as feasibility and costs.

### Brain state

Brain state is defined as the state the person is in when the TMS treatment is delivered. We separately discuss contextual cues that influence the immediate state when TMS is delivered and concurrent treatments that the patient receives throughout the course of TMS.

#### Context

One of the outstanding questions in the field is whether there is an interaction between the context of the brain (i.e., what an individual may be thinking or feeling) and effects of brain stimulation. There is research suggesting differential effects of TMS depending on the context [73]. Usually, TMS is delivered while the patient is at rest (i.e., not asleep, watching TV, listening to music, etc), however, a few studies have investigated the impact of changing the brain state during the session and measuring its effect. In an early study of 20 Hz TMS using the H1 TMS coil, Isserles et al. demonstrated that negative mood induction could attenuate the antidepressant effects of stimulation [74]. Cue provocation is another method that could change the context in which TMS is delivered and augment the efficacy of TMS. For example, cue provocation has been used to change the brain state before or during delivering TMS for smoking cessation [75, 76], though one study showed a placebo effect [77]. Similarly, symptom provocation before TMS is used in treatment of obsessive compulsive disorder (OCD) [78, 79]. The brain state could also be changed by combining TMS with medication. One example is the recent demonstration that d-cycloserine can improve response rates when used in combination with iTBS [80]. Other options include combined use of stimulation with various psychotherapy approaches, although whether and how to combine these modalities remains an unanswered question, with some evidence that the type and timing of the psychotherapy is likely to be important. For example, exposure plus stimulation for OCD appears to yield superior clinical outcomes [78], yet a similar approach for PTSD attenuated effects of active stimulation when using TMS with an H1 coil [81]. Although naturalistic studies indicate benefit of combined TMS plus psychotherapy for depression (e.g., ref. [82]), there are no prospective examinations comparing TMS versus TMS plus evidence-based psychotherapy.

#### Concurrent treatments

Given that TMS is commonly used for treatment-resistant depression and is offered after patients have failed at least one medication trial, most patients in clinical trials and receiving TMS in the community for MDD are on antidepressant medication(s). Whether and how medications influence clinical outcomes in accelerated TMS remains unclear. In one of the early randomized controlled trials of TMS monotherapy, clinical outcomes improved once participants were placed on antidepressant medications as they exited the trial, suggesting synergy [2]. While several medications (e.g., antiepileptic agents, benzodiazepines, psychostimulants, etc.) may impact motor threshold calibration, whether these medications reliably impact clinical outcomes in standard TMS remains unclear [83, 84]. Uncontrolled data has indicated reduced effectiveness for MDD when patients are on anticonvulsants, benzodiazepines, or antipsychotics (e.g., refs. [85–87]), and possible improved outcomes when on psychostimulants; [87, 88] yet the uncontrolled nature of these studies makes it difficult to ascertain whether these effects are directly related to the medication in question or are related to other clinical factors such as increased comorbidity. Of note, the SNT protocol allowed for anticonvulsant medication and only one (out of 14) patient in the active iTBS group and four (out of 15) in the sham group were not taking any other medications. This further underscores the need to understand the potential interactions (positive or negative) between stimulation and psychotropic medication, with the understanding that these interactions may be similar to or different from lessons learned from standard TMS.

#### Summary – Brain state

Brain state is likely a critical and complex factor in TMS treatment and research. Most RCTs and clinical practice use rest as the standard context and stable medication use as acceptable concurrent treatment, but effect of TMS may be different depending on the brain state. TMS could possibly be augmented (or impaired) by changing the context using provocation techniques, medication, or psychotherapeutic approaches. However, there is considerable nuance in this space that will require careful consideration; controlling brain state sounds laudable, but the actual procedures are likely to be unfeasible. For example, will studies need to find a way to have their participants maintain a single thought or series of thoughts during stimulation (or during an entire course of TMS)? An individual’s internal processes during tasks and provocation are also likely to vary and will need to be assessed or evaluated. Furthermore, depending on the type of concurrent medication use, clinical outcomes may be improved or hampered, but controlled studies are needed to establish any effects. Taken together, considering ways to control (and measure) brain state is a critical challenge in accelerated TMS and the field more broadly; investigators should expect the unexpected when navigating the intersection of an unknown brain state and higher cumulative TMS exposure during accelerated TMS.

## The promise and pitfalls of accelerated TMS

Early studies provided initial support that accelerated TMS is effective with improved treatment outcomes in depression compared to sham [50], but relatively few studies have directly compared the effects of accelerated TMS to standard TMS protocols in terms of efficacy, safety, and tolerability [89]. Preliminary data point to the potential that accelerated TMS can deliver an increased number of pulses in a shorter time to shorten the response time in depression over standard protocols. However, it remains unclear how many treatments a day are optimal, what is the most efficient intertrain duration, and what is the maximum number of pulses that can be administered safely.

Even basic questions such as the durability of accelerated TMS have not been answered. The possibility that the speed of acute antidepressant response in studies of rTMS may be inversely related to the likelihood of relapse has been postulated for some time (e.g., ref. [90]). There are limited studies of the durability of TMS (e.g., ref. [91, 92]), and early data from existing accelerated TMS studies indicate a more rapid loss of acute efficacy [42, 43, 64]. More research is needed in this area. Ameliorating this concern somewhat is the fact that TMS has a high rate of re-response when administered subsequently [92] which was also suggested in a very small number (*n* = 6) of retreated patients described in Cole et al. [43].

Accelerated approaches may yield additional safety concerns particularly if the number of treatments or pulses is increased to further improve the response time. To date, the safety profile of TMS has been favorable, given its absence of the systemic side effects, and small risk of seizure, but it is reasonable to expect a different safety profile as doses increase over time. At this point in the research on accelerated protocols it will be important to develop systematic methods to assess in real time the individual’s cortical excitability (i.e., perhaps by EEG measures) in relation to the stimulation protocol. This can help determine the range of individual variability. E-Field modeling can also help determine the applied intensity of neurostimulation parameters based on an individual’s neuroanatomy, potentially increasing the stimulation above the standard maximum stimulus of 120% of the motor threshold. Assessing cortical excitability can add a measure of safety with the potential for increased stimulus parameters.

Incidence of treatment-emergent mania with iTBS protocols [93] and anxiety with standard TMS therapy has been reported (e.g. refs. [43, 94]), and as such should remind clinicians to carefully monitor patients receiving accelerated treatments. Safety will also have to be evaluated when applying accelerated approaches to different patient populations that may have different risk profiles, such as pediatric and geriatric patients. Similarly, patients with comorbid substance use and other common clinical conditions that place them at higher risk of seizure will need to be carefully studied.

Access to accelerated TMS must also be considered. Technologies need to be scaled to use by nonacademic practitioners if they are determined to be essential to the safety and efficacy of the accelerated treatments. Accelerated protocols are already being adopted into routine clinical practice even prior to studies to define the most essential elements of accelerated TMS. For example, are ten treatments a day required, or can that be shortened to eight treatments to conform to an eight-hour workday for a TMS administrator? And, is neuronavigation necessary to maximize response and how does it compare to more traditional methods such as the Beam/F3 method?

Of note, there have been attempts to develop at home TMS using lower risk devices to improve access. One example is the use of synchronized TMS, where low-field stimulation is synchronized to an individual’s alpha peak frequency [95]. However, multisite randomized controlled trials have reported mixed findings for the efficacy of low-field synchronized TMS in treating depression [96, 97]. Importantly, none of these trials have involved iTBS or accelerated protocols, which potentially increase the risks of side effects or serious adverse events. The safety of accelerated TMS should be determined before trials using self-administered devices.

Neuroimaging methods to identify a potential “ideal” location of stimulation have garnered significant attention over the last decade [67]. In fact, anticorrelation between the sgACC and TMS target has emerged as one of the most promising predictors of TMS response and was integrated into more recent studies of accelerated TMS for depression. While studies identifying TMS targets have shown positive preliminary results, it is unclear what the actual added benefit of this technique is, which is critical to understand given the additional costs and burden. There are several important unknowns regarding this approach and more mechanistic research in this area is imperative. Predictors for TMS treatment success, such as greater pre-treatment functional or structural connectivity are needed to further optimize personalization. Moreover, it is unclear what the role of structural versus functional brain connections is in defining the TMS treatment target. A recent study demonstrated the importance of overlap between structural and functional connectivity in enhancing the impact of TMS [98], which suggests future studies should also consider structural connections in defining the TMS target, yet all clinical studies so far have used structural targets or functional connectivity targets, not taking into account structural connections between regions.

One of the key challenges in clinical TMS is that patients must fail several antidepressant treatments to be approved for commercial insurance coverage of their course of TMS. An alternative strategy would be to implement a stratified or precision medicine approach; using this framework, a patient would undergo a biomarker assessment that would provide treatment-specific guidance for the patient and clinician. In a study nearly a decade ago, McGrath et al. [99], demonstrated that brain glucose metabolism identified patients more likely to respond to cognitive behavioral therapy versus an antidepressant. In the last several years, multiple clinical investigations demonstrated the potential of biomarkers to predict antidepressant treatment response, which included central and autonomic nervous system regulation [100], electroencephalogram [101–103], fMRI functional connectivity [104, 105], and subcortical volumes [106]. In the example of accelerated TMS, measurement of a TMS-specific biomarker, obtained after the initial diagnosis of depression or first antidepressant failure, would inform whether to pursue TMS (e.g., ref. [107]). Additionally, there is a need for biomarkers that could predict efficacy of TMS versus antidepressants (such as [108]) or cognitive behavioral therapy. This could be an important future strategy for earlier stratification of patients who would be good candidates for TMS and supports the potential of TMS as a first-line treatment option if supported by biomarker data. This process, however, requires TMS-response biomarkers to be reliable and specific; at the current time no biomarkers have demonstrated sufficient validity for clinical use, although several hold promise and are under active development [109].

Furthermore, the field should also expect that some of the prior lessons learned may come with crucial caveats. As an example, recent examinations have raised important new questions about the use of personalized sgACC-dlPFC functional targeting. An analysis of imaging data from Blumberger et al. [38] identified that this connectivity relationship poorly predicted treatment response and appeared to be heavily influenced by what appears to be respiratory artifact [69]. While the field has yet to consider the full impact of this work, it should remind researchers to carefully examine of core components of clinical response.

To date, neuroimaging studies have largely focused on comparing fMRI measures before and after treatment. However, these designs provide limited direct information on how the brain changes during the course of stimulation. Repeatedly, early clinical response to TMS has broadly predicted longer-term outcomes ([110], but also see [111]). Recently, work from Berlow et al. [112] indicated that TMS response follows an exponential decay function, regardless of the protocol or approach (e.g., accelerated vs. standard). Assuming a relationship between brain changes and clinical symptom change, this indicates that large-scale changes in circuit reorganization may be occurring early in the TMS course, and empirically argues for oversampling (e.g., using MRI, EEG) during the first weeks of TMS to characterize these large-scale changes [113]. Once these early changes have been identified, different stimulation approaches can be designed to provide a maximally accelerated response. There is also a need for studies investigating the immediate effect of such approaches on brain function and functional connectivity. A few mechanistic studies using interleaved TMS-fMRI have provided first evidence for a causal relation between the DLPFC stimulation and activation in the sgACC and its broader functional network, though findings were not all in the same direction likely due to differences in study designs [114–116], suggesting the need for more research in this area.

It seems ideal to maximize treatment in a short amount of time, but patients must be able to spend a full week to undergo the treatment and the clinic schedule must be able to accommodate this as well. By its definition, this is an expensive intervention combined with further expensive approaches (e.g., EEG, MRI, neuronavigation, etc.), with additional costs related to staffing. Whether these interventions can be implemented in resource-poor environments is also an important consideration. At the crux of this question is: Are these more expensive approaches superior? The field is overdue for properly powered and conducted studies comparing high cost technical TMS approaches versus lower cost interventions, or even comparisons between higher cost TMS and earlier forms of TMS. If the effect sizes are truly superior with accelerated approaches, then the increased short-term cost to reduce longer-term and well-documented impacts of depression can be justified. If, however, the gains are more modest, then the field will need to carefully consider how and where to deploy accelerated TMS (e.g., inpatient treatment while hospitalized), and use the knowledge gained from high-cost approaches and translate them into lower cost interventions with broader and more equitable reach.

## Future research directions and clinical implications

Accelerated TMS is a promising application of TMS that increases the number of TMS pulses that can be delivered, reduces treatment time, and achieves a more rapid reduction in depressive symptoms. However, the literature supporting this approach remains at a very early stage, and it is possible, and even likely, that misinterpreting efficacy signals from interrelated variables will lead us to miss important signals hidden within our data. Precisely which of these nine elements is critical and what parameters for each of the elements is most optimal for the treatment of MDD remains unclear at this time. To this end, there are ongoing research initiatives that will evaluate different elements of accelerated TMS. Yet, it is important to recognize these studies will still depend upon imprecise measures of self- or clinician-assessed symptom severity measures, and so these studies must be coupled with biological measures (fMRI, EEG, etc.). Several large-scale studies are ongoing [113] to determine how neurophysiologic outcomes can be used to optimize treatment parameters and assess therapeutic efficacy, and adopting a high sampling of biological signals early in the treatment course remains a promising (and relatively unexamined) area of inquiry that can be integrated into existing treatment protocols. Taken together, the field is at an important crossroads, with new data indicating that the promise of accelerated clinical outcomes, without systemic side effects, may be within our reach. Yet, we must be mindful that early adoption can come at the price of missing opportunities to further develop accelerated TMS. And finally, as future studies progress it will be incumbent upon the field to ensure that the resultant treatments remain accessible to the patients who need them the most.
