# Correction: Accelerated TMS - moving quickly into the future of depression treatment

**DOI:** 10.1038/s41386-023-01714-0

**Published:** 2023-08-28

**Authors:** Sanne J. H. van Rooij, Amanda R. Arulpragasam, William M. McDonald, Noah S. Philip

**Affiliations:** 1 Emory University School of Medicine, Department of Psychiatry and Behavioral Sciences, Atlanta, GA USA; 2 Alpert Medical School of Brown University, Department of Psychiatry and Human Behavior, Providence, RI USA; 3 VA RR&D Center for Neurorestoration and Neurotechnology, VA Providence Healthcare System, Providence, RI USA

**Keywords:** Outcomes research, Depression, Depression

Correction to: *Neuropsychopharmacology* 10.1038/s41386-023-01599-z, published online 22 May 2023

In the original article, the description of the accelerated iTBS protocol by Cole et al. [42, 43] contained some errors. In the sub-section ‘Cumulative exposure’, the following sentences were amended. The sentence “The largest reported number of pulses per session is the protocol described by Cole et al. [42, 43]. In this protocol, 9000 pulses are delivered per session for ten sessions per day, totaling 90,000 pulses per day, providing evidence that this high number of pulses with 50-min delays between sessions can be delivered safely” was corrected to “The largest reported number of pulses per session is the protocol described by Cole et al. [42, 43]. In this protocol, 1800 pulses are delivered per session for ten sessions per day, totaling 18,000 pulses per day, providing evidence that this high number of pulses with 50-min delays between sessions can be delivered safely”. The sentence “Extant studies have shown that delivering as much as ten sessions per day with a total of 90,000 pulses for five consecutive days appears safe from early pilot randomized controlled trials” was corrected to “Extant studies have shown that delivering as much as ten sessions per day with a total of 18,000 pulses for five consecutive days (90,000 pulses in total) appears safe from early pilot randomized controlled trials”. These corrections do not change the conclusions of the article. The original article has been corrected.

